# Distal migration of a floating carotid thrombus in a patient using oral contraceptives: a case report

**DOI:** 10.4076/1752-1947-3-8389

**Published:** 2009-07-14

**Authors:** Masaki Watanabe, Takahisa Mori, Keisuke Imai, Hajime Izumoto, Teruyuki Hirano, Makoto Uchino

**Affiliations:** 1 Department of Stroke Treatment, Shonan Kamakura General Hospital, 1202-1 Yamazaki, Kamakura, Kanagawa, Japan; 2 Department of Neurology, Graduate School of Medical Sciences, Kumamoto University School of Medicine, 1-1-1 Honjo, Kumamoto, Japan

## Abstract

**Introduction:**

We report the case of a patient with distal migration of a floating carotid thrombus caused by oral contraceptives.

**Case presentation:**

A 48-year-old woman using oral contraceptives suffered from dysarthria and gait disturbance. Examinations, including ultrasound and cerebral arteriogram, revealed a floating thrombus at the left carotid bifurcation with no stenosis. Despite antithrombotic therapy, the floating carotid thrombus migrated to the ipsilateral middle cerebral artery, resulting in a severe stroke.

**Conclusion:**

Some floating thrombi are resistant to conservative therapy and have a risk of distal migration, which may cause a major stroke in the acute stage.

## Introduction

Carotid endarterectomy (CEA) is the standard treatment for extracranial carotid occlusive diseases. However, determining a more appropriate therapy, surgery or medical management, for treating a floating thrombus in the carotid artery is controversial. Some reports have recommended conservative therapy such as antithrombotic drugs [1,2], while others have diagnosed the floating thrombus as a critical lesion and recommended aggressive therapy, including emergent CEA [3]. In this report, we describe the distal migration of a floating carotid thrombus to the ipsilateral middle cerebral artery despite intensive antithrombotic therapy.

## Case presentation

A 48-year-old Asian woman with dysmenorrhea and taking oral contraceptives (Edulen, ethinylestradiol 50 μg, ethynodiol acetate 1000 μg) for 6 months suddenly developed difficulty in speech at 7 p.m. on 25 September 2003. Her symptoms resolved in a few minutes. A re-attack of the difficulty in speech and unsteadiness in walking occurred on October 1, and she was admitted to our hospital. Her mother and grandfather had a medical history of cerebral infarction; however, the details were unclear. The patient had been a cigarette smoker (20/day) since 1999.

A physical examination revealed no disturbance of consciousness or cognitive dysfunction, but mild weakness in her right leg was demonstrated. The patient was assessed and was graded with a score of 2 on the National Institutes of Health Stroke Scale (NIHSS). An immediate brain computed tomography (CT) demonstrated no early ischemic changes; however, diffusion-weighted images (DWI) on magnetic resonance image (MRI) showed a slightly high intensity lesion along the left insular cortex. No laterality was detected in the perfusion-weighted MR images (PWI). MR angiography displayed poor visualization of the branches of the left middle cerebral artery (MCA).

**Figure 1:**
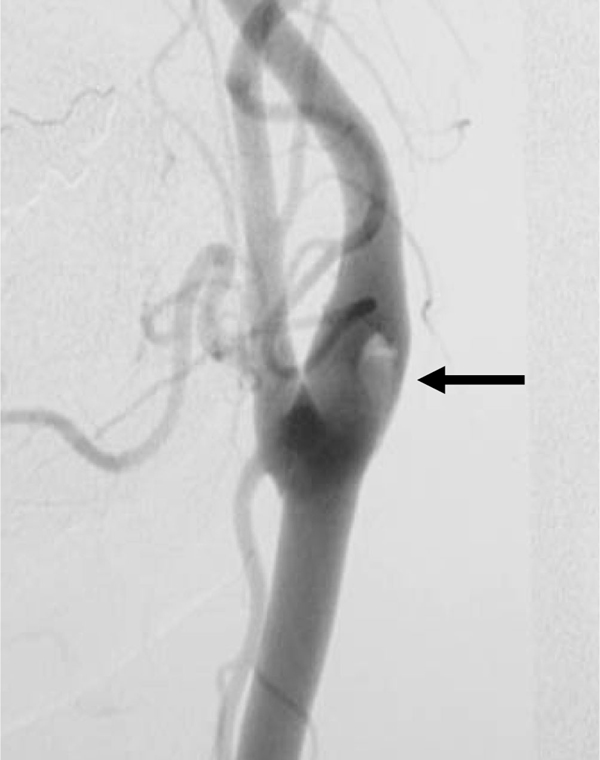
**Cerebral angiogram performed on 1 October 2003**. The floating thrombus is observed at the left internal carotid artery (arrow).

**Figure 2:**
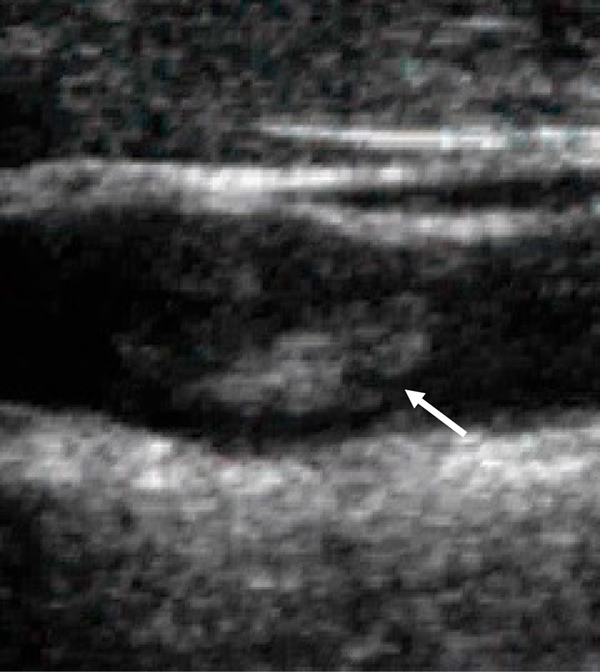
**Carotid duplex sonography performed on 1 October 2003**. The floating thrombus is observed at the left internal carotid artery (arrow).

Cerebral angiograms disclosed a floating thrombus at the left carotid bifurcation (Figure 1). A carotid duplex sonography with a GE LOGIQ 700 showed a 3 mm isoechoic floating thrombus originating from the antero-lateral carotid wall (Figure 2). The floating thrombus appeared to move slightly with the cardiac cycle. Significant atherosclerotic change of underlying intima-media could not be seen at the left carotid artery. Both transthoracic and transesophageal echocardiograms appeared normal. There was no arrhythmia detected by ambulatory electrocardiography. Routine biochemical and hematologic tests, including prothrombin time, partial thromboplastin time, platelet count, anti-thrombin III, protein C, protein S, anti-nucleotide antibody and anti-cardiolipin antibody, were within normal limits. Cerebrospinal fluid analysis showed no abnormality.

**Figure 3:**
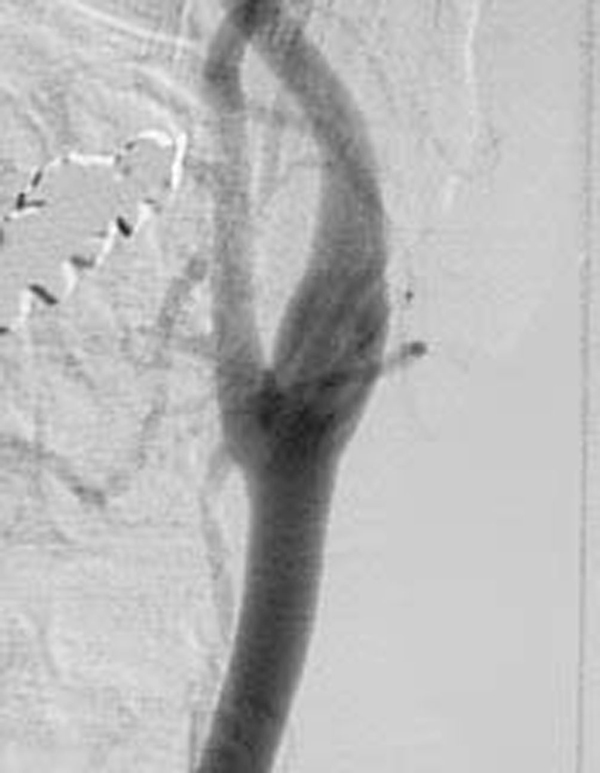
**Cerebral angiogram performed on 5 October 2003 just after neurological deterioration**. The floating thrombus is not observed.

**Figure 4:**
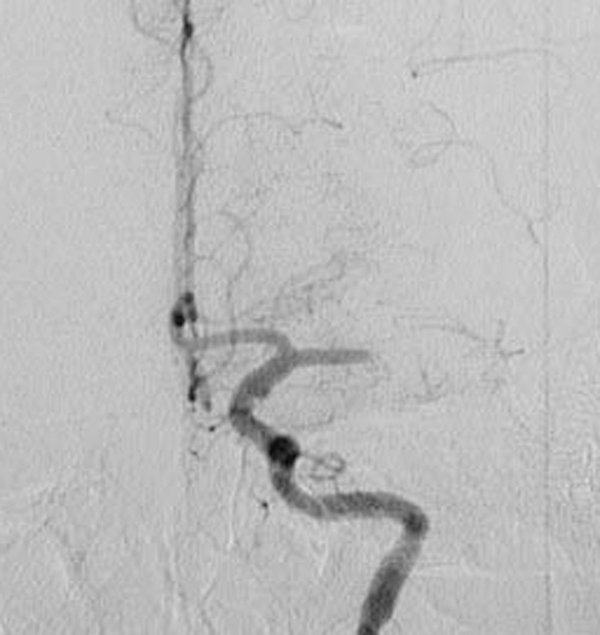
** Total occlusion of the horizontal portion of the left middle cerebral artery is clear on emergent cerebral angiogram**.

In spite of the hyper-acute stage of ischemic stroke, we determined to treat her not by thrombolysis, but conservatively, due to the slight neurological deficit, no diffusion-perfusion mismatch, no arteriographic carotid stenosis and the peripheral branch occlusion of the MCA. We administered intravenous heparin (10,000 U/day), oral aspirin (100 mg) and ticlopidine (100 mg). Fortunately, her neurological deficits gradually improved and completely diminished in a few days. However, she suddenly exhibited total aphasia and severe right-sided hemiparesis 5 days after admission. MRI performed just after the ictal event documented a large diffusion-perfusion mismatch in the left MCA territory, and MR angiography revealed a total occlusion of the left MCA trunk. An emergent cerebral arteriogram demonstrated the disappearance of the carotid floating thrombus (Figure 3), no carotid stenosis and a total occlusion of the horizontal portion of the ipsilateral MCA (Figure 4).

Local intra-arterial thrombolysis followed by percutaneous transluminal cerebral balloon angioplasty was performed. Although partial recanalization of the left MCA was achieved, the large territorial infarct in the upper division of the left MCA was complete, causing severe neurological deficit (NIHSS score 21 on day 30). Follow-up carotid duplex sonography showed the disappearance of the floating thrombus and no plaques. Aspirin (100 mg) was continued as a drug of secondary prevention, and cigarette smoking and oral contraceptives were terminated. CEA was considered to be not indicated because there was no carotid residual stenosis.

## Discussion

A floating thrombus is not commonly identified, and previous angiographic studies have indicated that it is present in 0.4% to 1.5% of cases of ischemic cerebrovascular disease [1,4,5]. Most floating thrombi are associated with atheromatous plaques, cardiogenic emboli, arterial dissections and systemic diseases related to coagulopathic states [1,2,5,6]. Because the patient had neither carotid atheromatous plaques nor systemic illnesses that contributed to clot formation, such as collagen disease, systemic cancer and coagulation abnormalities, we concluded that the floating thrombus was associated with oral contraceptive use and cigarette smoking. Observation with a scanning electron microscope has often documented ultramicroscopic ulcerations and thrombi, even in smooth appearing plaques [7]. Oral contraceptives and smoking may activate the coagulation cascade in the carotid bifurcation where the arterial intima tends to be disrupted by turbulent flow.

An association between cerebral infarction and the use of contraceptives, especially with smoking and a history of hypertension, was established previously [8]. However, reports linking contraceptives and floating thrombi are rare. Buchan *et al.*[1] described an intraluminal thrombus associated with contraceptives, and reported that the thrombus was diminished by treatment with heparin and warfarin. However, the clinical course of our patient should alert clinicians that some floating thrombi are resistant to conservative therapy and have a risk of distal migration that may cause a major stroke in the acute stage.

An intensive antithrombotic therapy is generally considered a first line management option in treating a floating thrombus [1,2]. Surgical intervention is considered a secondary treatment plan, even with the presence of carotid stenosis in the chronic stage. On the other hand, Goldstone and Moore [3] adopted emergent CEA for the treatment of acute unstable stroke patients with floating thrombi, defining these thrombi as critical arterial lesions. Biller *et al.*[4] advocated surgical therapy if the intraluminal clots of the carotid artery involved an accessible lesion in a patient with progressive stroke despite medical therapy. Progressive stroke in patients with a floating thrombi coupled with a high-grade internal carotid artery (ICA) stenosis after starting conventional treatment have been documented [9]. The authors proposed an optional surgical intervention of carotid artery stenting (CAS) with filter-type devices. Another cerebral protection device consisting of an inversion of ICA blood flow that is achieved by balloon occlusion of the common carotid artery (CCA) and external carotid artery (Parodi antiembolism system) may have more benefits than CAS for such diseases [10].

Because no stenotic lesion was observed in the carotid artery of our patient, surgical intervention was not considered to be a suitable option. Dual antiplatelet therapy along with anticoagulation resulted in an unfavorable outcome. We adopted fixed-dose heparin administration and did not measure activated partial thromboplastin time before the recurrence of the stroke. Therefore our medical management might possibly have been insufficient therapy. Further investigations are required to determine which course of treatment, medical intervention, surgical intervention or a combination, is suitable for patients with a floating carotid thrombus and the presence or absence of carotid stenosis.

## Conclusion

The more appropriate therapy, surgery or medical management, for treating a floating thrombus in the carotid artery is still controversial. This case report highlights that some floating thrombi in the carotid artery are resistant to conservative therapy and have a risk of distal migration that may cause a major stroke.

## Abbreviations

CAS: carotid artery stenting; CCA: common carotid artery; CEA: carotid endarterectomy; CT: computed tomography; DWI: diffusion-weighted images; ICA: internal carotid artery; MCA: middle cerebral artery; MRI: magnetic resonance image; NIHSS: National Institutes of Health Stroke Scale; PWI: perfusion-weighted magnetic resonance images.

## Consent

Written informed consent was obtained from the patient for publication of this case report and any accompanying images. A copy of the written consent is available for review by the Editor-in-Chief of this journal.

## Competing interests

The authors declare that they have no competing interests.

## Authors' contributions

All authors have contributed equally and have given approval of the version to be published.
